# Little impact of tsunami-stricken nuclear accident on awareness of radiation dose of cardiac computed tomography: A questionnaire study

**DOI:** 10.1186/1756-0500-6-170

**Published:** 2013-04-30

**Authors:** Sung Hea Kim, Hyun-Joong Kim, Hyun Kyun Ki, Eui-Jong Chung, Soon Yong Suh, Seong Woo Han, Kyu-Hyung Ryu

**Affiliations:** 1 Department of Internal Medicine, Konkuk University School of Medicine, 1, Hwayang-dong, Gwangjin-gu, Seoul, South Korea; 2 Heart Center, Gachon University of Medicine and Science, Gil Hospital, Incheon, South Korea; 3 Department of Internal Medicine, College of Medicine, Hallym University, Seoul, South Korea; 4 Division of Cardiology, Department of Internal Medicine, College of Medicine, Hallym University Dongtan Sacred Heart Hospital, 40, Sukwoo-Dong, Hwaseong-Si, Gyeonggi-Do 445-170, South Korea

**Keywords:** 64-channel MDCT, Radiation dose, Questionnaires, Awareness

## Abstract

**Background:**

With the increased use of cardiac computed tomography (CT), radiation dose remains a major issue, although physicians are trying to reduce the substantial risks associated with use of this diagnostic tool. This study was performed to investigate recognition of the level of radiation exposure from cardiac CT and the differences in the level of awareness of radiation before and after the Fukushima nuclear plant accident.

**Methods:**

We asked 30 physicians who were undergoing training in internal medicine to determine the equivalent doses of radiation for common radiological examinations when a normal chest X-ray is accepted as one unit; questions about the absolute radiation dose of cardiac CT data were also asked.

**Results:**

According to the results, 86.6% of respondents believed the exposure to be 1 mSv at most, and 93.3% thought that the exposure was less than that of 100 chest X-rays. This finding indicates that their perceptions were far lower than the actual amounts. Even after the occurrence of such a large nuclear disaster in Fukushima, there were no significant differences in the same subjects’ overall awareness of radiation amounts.

**Conclusions:**

Even after such a major social issue as the Fukushima nuclear accident, the level of awareness of the accurate radiation amount used in 64-channel multidetector CT (MDCT) by clinical physicians who order this test was not satisfactory. Thus, there is a need for the development of effective continuing education programs to improve awareness of radiation from ionizing radiation devices, including cardiac CT, and emphasis on risk-benefit evaluation based on accurate knowledge during medical training.

## Background

With recent advances in technology, the importance of cardiac imaging has also increased. In particular, the roles of both cardiac computed tomography (CT) and magnetic resonance imaging (MRI) have expanded. Cardiac CT, because of its advantage of allowing for noninvasive evaluation of both coronary artery stenosis and myocardial perfusion, has been suggested as a substitute tool for conventional coronary angiography in the early detection of ischemic cardiac disease [1,2]. Although recent reports have recognized its limitation in replacement of conventional coronary angiography in terms of diagnostic accuracy of coronary artery diseases, the frequency of use of cardiac CT in clinical practice is gradually increasing [3,4].

However, unlike MRI, cardiac CT has an innate issue with regard to its radiation hazard, which is known to be higher than that of other CT modalities because of cardiac motion. For proper use of cardiac CT, clinicians must have accurate knowledge of not only its advantages, but also its limitations, especially with regard to radiation hazard. Thus, awareness of radiation hazard by healthcare professionals is essential and important for proper evaluation of the risk-benefit ratio.

The aim of this study was to investigate medical residents’ awareness of the radiation dose used in the performance of cardiac CT and assess the impact of social issues, such as the Japan nuclear plant explosion, on the level of awareness.

## Methods

The IRB exempted survey was conducted from November 2009 to March 2010 as part of the training and assessment of medical residents (KUH 1010479). The survey aimed to improve their perceptions of the absolute radiation dose in one prescription of 64-channel multidetector (MDCT) and the relative radiation dose compared with that of a simple chest PA. The subjects included 30 medical residents who were undergoing training in internal medicine. An earthquake subsequently occurred in Fukushima, Japan in March 2011. A follow-up survey was performed on the same subjects using the same questionnaire. The change in the degree of awareness of pre- and post-earthquake radiation exposure was evaluated. Research carried out in compliance with the Helsinki Declaration.

The survey sheet included two categories: the general characteristics of the respondents and the awareness of the exposure by each test. Regarding awareness of radiation exposure, the first survey asked questions on radiation doses for conventional angiography, cardiac MDCT, and MIBI scan compared with a simple chest PA to determine the relative radiation dose. In the second questionnaire, subjects were asked to determine the absolute radiation dose for cardiac MDCT (Additional file 1: Figure S1).

**Figure 1:**
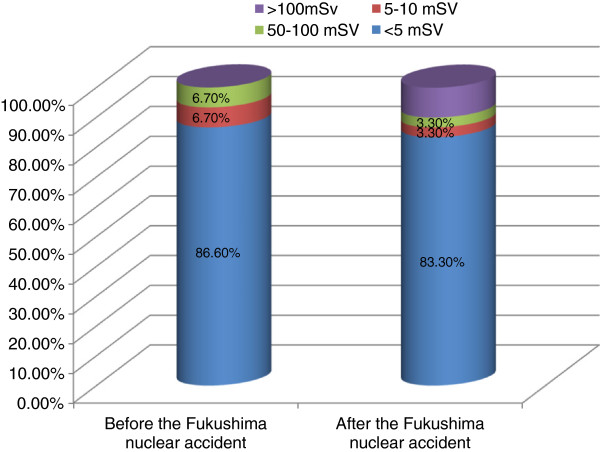
Percent of subjects according to the perceived absolute radiation amount of Cardiac CT before and after nuclear accident.

Numerical variables are shown as mean ± standard deviation or median (minimum, maximum) values, and categorical variables are shown as number and percentage. The Kolmogorov-Smirnov test was used to determine normal distribution. Nonparametric statistics, including the Wilcoxon rank sum test, were employed for comparison of differences in variables between the first and second survey; p < 0.05 was considered statistically significant. PASW statistics software (version 17) was used to perform the statistical analysis.

## Results

The mean age of the subjects was 31 ± 2.5 years, and 19 subjects (63.3%) were male. The median value of the absolute radiation exposure was 1 mSv (0.5 mSv, 100 mSv) on the first survey and 1 mSv (0.1 mSv, 300 mSv) on the second survey. According to the results of the Wilcoxon rank sum test, there was no difference between the first and second survey (z = −1.483, p = 0.138).

On the first survey, the median value of the relative radiation exposure of cardiac CT compared with a simple chest X-ray was 50 times (10, 500), which did not differ from that of the second survey (50 times [10, 1000]) (z = −4.47, p = 0.655).

**Figure 2:**
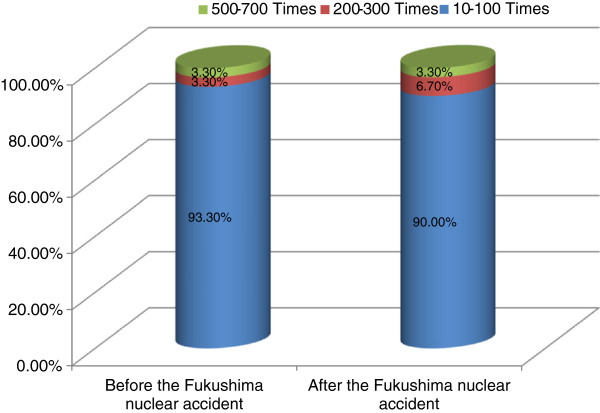
Percent of subjects according to the perceived relative radiation amount of Cardiac CT before and after nuclear accident.

The 64-channel MDCT is generally known to carry a radiation exposure of 5 to 10 mSv per shot, which is equivalent to 300 times the amount of a simple chest X-ray. However, 86.6% of the respondents on the first survey and 83.3% on the second survey perceived the exposure to be 1 mSv at most. On the first and second surveys, 93.3% and 90.0% of respondents thought that the exposure was 100 times less than the amount in the chest X-ray, respectively (Figures 1 and 2).

## Discussion

In our study, we found that medical residents’ level of knowledge of the radiation dose of cardiac CT is low and inadequate. However, previous studies have highlighted the lack of appreciation of radiation doses, even in radiologists [5-7]; therefore, this result is not surprising.

Second, there was no improvement in the clinicians’ degree of awareness regarding radiation exposure between the pre- and post-earthquake periods, despite the experience of such a major social issue and the resulting information campaign about exposure through newspapers and television for several weeks, which caused even laymen to become familiar with the international unit of radiation dose (the sievert). This may suggest that the clinicians believed that their underestimated radiation dose was adequate and not in need of correction, even after the nuclear plant accident, which indicates a fixed underestimated status.

As the clinical areas of CT expand, the frequency of use of CT and concerns about increases in the exposure dose are likewise increasing. However, even a small exposure dose could be hazardous, as seen during the Hiroshima atomic bombing [8,9]; therefore, clinicians should make every effort to minimize the radiation dose.

To decrease the radiation dose from CT equipment used in cardiac MDCT, efforts have been made to control the amount of X-ray by the attenuation level or to decrease the amount of X-ray at the systolic phase of electrocardiography (ECG); these efforts have resulted in some verifiable success. One study reported on a trial to decrease the radiation exposure by up to 5 mSv using a low-dose coronary CT technique with 100 kVp rather than 120 kVp; however, the diagnostic accuracy of this technique has not yet been verified [10]. Various strategies to decrease the radiation dose in coronary CT angiography or coronary calcium CT have been applied, including the use of low amperage, low voltage, and ECG-triggered amperage modulation. In particular, ECG-triggered amperage modulation has been reported to lower the effective dose by 20% to 50% based on the heart rate [10-13]. However, prior to the establishment of technical advancements, improvements in clinicians’ awareness and knowledge of radiation should occur, because proper knowledge regarding risk is the first step in risk-benefit analysis in our practice.

There could be several explanations for the low level of clinicians’ awareness of radiation. Clinicians’ focus on treatment methods and the relative indifference to the test method itself may play a major role in the lack of understanding. Other reasons for the lack of understanding may include the image-centric thinking pattern of the modern generation, lack of systematic and repetitive education systems, and lack of knowledge of radiation hazard.

As demonstrated in this study, a one-time shock has no significant educational effect. Thus, systematic and repetitive education systems may be first to be established ultimately to increase awareness of the potential risk of radiation. Another way to increase awareness is visualization of total radiation doses using figures or graphics on its readings, not only on images, which may alarm clinicians to recall the risk and benefit once more [14].

### Study limitations

First, the number of subjects was small to represent all of trainee or medical residents. Second, our survey was not originally designed to compare the level of perceptions of radiation dose before and after social issue, rather to improve their perceptions of the radiation dose as part of the annual training program. Thus, the questionaires used in our survey may have not sufficient validity and reliability to evaluate the awareness of radiation dose. But, because there is no established tool of evaluating the level of perceptions of radiation dose, the recent studies have to depend on questionnaire survey [15-20].

## Conclusions

The results of our study highlight that the level of awareness of the accurate radiation amount used in the performance of 64-channel MDCT by clinical physicians who order the test is not satisfactory. Therefore, it is suggested that clinical physicians can safely perform multiple-channel MDCT on patients not only through being properly informed on the use of multiple-channel MDCT, but also through active training on the possible risk of patients’ exposure. In addition, development of an effective educative method and the optimum method for improvement of awareness is needed.

## Competing interests

The authors declare that they have no competing interests.

## Authors’ contributions

SHK and EJC participated in data analysis and drafted the initial manuscript. HJK and SYS participated in the sequence alignment and critical revision. HKK and SWH participated in the design of the study and performed the statistical analysis. KHR conceived the study, participated in its design and coordination, and helped to draft the manuscript. All authors made substantial contributions to the acquisition of data. All authors read and approved the final manuscript.

## Supplementary Material

Additional file 1: Figure S1 Key questionnaires used in the Survey Click here for file
