# Supplementary material for: Little impact of tsunami-stricken nuclear accident on awareness of radiation dose of cardiac computed tomography: A questionnaire study
Source: BMC Res Notes. 2013 Apr 30;6:170. doi: 10.1186/1756-0500-6-170 (PMC3682884; doi:10.1186/1756-0500-6-170)
Supplement: Additional file 1: Figure S1 — Key questionnaires used in the Survey [file 1756-0500-6-170-S1.pdf]

Q1) How much more radiation dose do the following tests have compared with chest X-ray?

Conventional angiography ( ) times more

Cardiac MDCT ( ) times more

MIBI scan ( ) times more

Q2) Please list the following tests in the descending order of radiation dose

1) Conventional angiography

2) Cardiac MDCT

3) MIBI scan

4) Radiation dose to which a person is naturally exposed in the environment in one year

Q3) How much is the average radiation dose of one cardiac CT scan. Answer in Sievert unit (mili).

Cardiac MDCT ( ) mSv
